# Identification of *Borrelia* Species after Creation of an In-House MALDI-TOF MS Database

**DOI:** 10.1371/journal.pone.0088895

**Published:** 2014-02-12

**Authors:** Adriana Calderaro, Chiara Gorrini, Giovanna Piccolo, Sara Montecchini, Mirko Buttrini, Sabina Rossi, Maddalena Piergianni, Maria Cristina Arcangeletti, Flora De Conto, Carlo Chezzi, Maria Cristina Medici

**Affiliations:** Unit of Microbiology and Virology, Department of Clinical and Experimental Medicine, Faculty of Medicine and Surgery, University of Parma, Parma, Italy; University of Kentucky College of Medicine, United States of America

## Abstract

Lyme borreliosis (LB) is a multisystemic disease caused by *Borrelia burgdorferi sensu lato* (*sl*) complex transmitted to humans by *Ixodes* ticks. *B*. *burgdorferi sl* complex, currently comprising at least 19 genospecies, includes the main pathogenic species responsible for human disease in Europe: *B*. *burgdorferi sensu stricto* (*ss*), *B*. *afzelii*, and *B*. *garinii*. In this study, for the first time, MALDI-TOF MS was applied to *Borrelia* spp., supplementing the existing database, limited to the species *B*. *burgdorferi ss*, *B* **.** *spielmanii* and *B*. *garinii*, with the species *B*. *afzelii*, in order to enable the identification of all the species potentially implicated in LB in Europe. Moreover, we supplemented the database also with *B*. *hermsii*, which is the primary cause of tick-borne relapsing fever in western North America, *B*. *japonica*, circulating in Asia, and another reference strain of *B*. *burgdorferi ss* (B31 strain). The dendrogram obtained by analyzing the protein profiles of the different *Borrelia* species reflected *Borrelia* taxonomy, showing that all the species included in the *Borrelia sl* complex clustered in a unique branch, while *Borrelia hermsii* clustered separately. In conclusion, in this study MALDI-TOF MS proved a useful tool suitable for identification of *Borrelia* spp. both for diagnostic purpose and epidemiological surveillance.

## Introduction

Lyme Borreliosis (LB) is a multi-systemic inflammatory disorder caused by an immune response to the pathogenic species of a group of closely related spirochetes, *Borrelia burgdorferi sensu lato* (*sl*) complex, that is sustained mainly by wild animals and transmitted to humans by hard ticks belonging to the genus *Ixodes* [1]–[3]. The infection results in a wide range of clinical manifestations that may affect skin, joints, heart and nervous system [4]–[7].

*B. burgdorferi* complex, currently comprising at least 19 different taxonomic entities or genospecies [8], includes the main pathogenic species responsible for human disease in Europe: *B*. *burgdorferi sensu stricto* (*ss*), the most arthritogenic, *B*. *afzelii*, mostly associated with skin manifestations, and *B*. *garinii*, the most neurotropic [3], [9]. Among the other species, *B*. *spielmanii* has been detected in early skin disease, *B*. *bissettii* and *B*. *valaisiana* have been detected in specimens from single cases of LB; the clinical role of *B*. *lusitaniae* remains to be substantiated [10]. *B*. *hermsii* is the primary cause of tick-borne relapsing fever in western North America [11]. Other species are not pathogenic to man, such as the Japan-specific *B*. *japonica* [12], *B*. *tanukii* and *B*. *turdi* [13], and the America-specific *B*. *andersonii* [14].

The spread of LB depends on geographical, environmental, and climatic factors. Epidemiological data about LB mostly derive from local/regional surveys. In Europe it has become an important infectious disease most prevalent in forested areas such as Scandinavia and Central Europe [1], [15], whilst in countries such as Italy it is a quite rare disease as also reported by us in our setting [1].

Although the diagnosis of LB is primarily based on the most obvious clinical sign, *erythema migrans*, the diagnosis of other forms of LB requires confirmation by microbiological assays [3], [16].

Among the direct diagnostic assays, culture from different clinical samples, such as skin, blood, and cerebrospinal fluid, is difficult because it is not useful for patients with *erythema migrans* and too insensitive for patients with extra-cutaneous manifestations of LB [16], [17]. However, the chance of isolating the strain is greatly increased if samples are taken at the primary phase of the illness, as early as possible after the first clinical signs are observed and when systemic symptoms are present. Thus, isolated strains can be subsequently identified [7], [16], [17]. The use of Polymerase Chain Reaction (PCR)-based methods increases the sensitivity considerably: after the first PCR assay reported in 1989 [18], various other PCR protocols were subsequently developed for the detection of *Borrelia* DNA in clinical specimens [1], [17], [19]–[21].

The complexity of the antigenic composition of *B*. *burgdorferi sl* and the temporal appearance of antibodies to different antigens at successive intervals of time after the infection implies that the development of a serological test with high sensitivity and specificity is a challenge [3]. Thus, serology remains of limited use because of its poor sensitivity at early stages of the disease and its lack of specificity at later stages [16]–[18].

In the last few years, matrix-assisted laser desorption ionization-time of flight mass spectrometry (MALDI-TOF MS) has become a powerful tool in the clinical microbiology setting and has revolutionized the work-flow also in our laboratory, enabling the rapid identification of bacteria and yeasts for clinical diagnosis [22]–[24]. The accuracy of this technique compares favourably with that of genomic sequencing and it is obtained at a significantly lower cost [22], [25].

However, no studies until now have been conducted about the application of MALDI-TOF MS to the identification of *Borrelia* spp. and the database of the mass spectrometer currently used in our laboratory for bacterial identification (MALDI Biotyper, Bruker Daltonics, Germany), nowadays one of the two commercially available instruments for microbiological identification of isolates to diagnostic purposes, is limited to the species *B*. *burgdorferi ss, B*. *garinii*, and *B*. *spielmanii*.

Even if in a recent survey [1] infection by *Borrelia* was revealed as rarely occurring in our area, in this study the already available MALDI-TOF MS database was supplemented with the missing species among those circulating in Europe, *B*. *afzelii*, obtaining an in-house reference database able to identify the overall species that are confirmed agents of localized, disseminated and chronic manifestations of LB in Europe, namely *B*. *burgdorferi ss, B*. *garinii*, and *B*. *afzelii* [10]. Moreover, other reference strains belonging to the *Borrelia* spp. collection available in our laboratory (*B*. *burgdorferi ss*, *B*. *hermsii*, and *B*. *japonica*) were tested. Finally, 6 additional field isolates of human and tick origin (belonging to the species *B*. *burgdorferi ss*, *B*. *garinii*, *B*. *afzelii*, and *B*. *lusitaniae*) were analyzed in order to provide evidence of the usefulness of MALDI-TOF MS for rapid, cheap and reliable identification of *Borrelia* strains at species level both for diagnostic and epidemiological purposes.

## Materials and Methods

### Ethics Statement

The *Borrelia* reference strains *B*. *afzelii* 103469, *B*. *hermsii* HS1, *B*. *japonica* F63B were provided from the French National Reference Centre for Borreliosis, Institut Pasteur, Paris, France. The *Borrelia* field isolates *B*. *burgdorferi* ZS7, *B*. *garinii* G1, *B*. *afzelii* FEM1-D15, *B*. *afzelii* ACA-1, and *B*. *lusitaniae* MT 0407-M8 were provided from the Institute of Medical Microbiology and Infection Control, Frankfurt, Germany. These strains, together with *B*. *burgdorferi ss* B31 (ATCC 35210) and the *B*. *burgdorferi ss* UCSC [26], [27], currently belong to our collection and are used in our laboratory for research use. Institutional Review Board approval was not required for the utilization of these strains, therefore anonymization of the strains was not applicable.

### Reference strains and field isolates

A total of 10 strains belonging to 6 different *Borrelia* species was included in this study: 4 reference strains, namely *B*. *afzelii* 103469, *B*. *hermsii* HS1, *B*. *japonica* F63B (all provided from Institut Pasteur, Paris, France), *B*. *burgdorferi ss* B31 (ATCC 35210), and 6 field isolates. These field isolates included: 1 strain (*B*. *burgdorferi ss* UCSC) belonging to a tourist from Venezuela and characterized in previous studies by molecular and conventional methods [1], [26]–[28] and 5 field isolates from different sources [29]–[31], namely *B*. *burgdorferi* ZS7 (tick isolate, Germany), *B*. *garinii* G1 (human cerebrospinal fluid isolate, Germany), *B*. *afzelii* FEM1-D15 (human skin isolate, Germany), *B*. *afzelii* ACA-1 (human skin isolate, Sweden), and *B*. *lusitaniae* MT 0407-M8 (tick isolate, Portugal).

All the strains were cultivated in Barbour, Stoenner, Kelly (BSK)-II medium (Sigma-Aldrich, Milan, Italy) supplemented with 6% rabbit serum (Sigma-Aldrich, Milan, Italy) at 30°C for 7 days [6], [16] for MALDI-TOF MS measurement. In order to verify the reproducibility of protein profiles, 2 different lots of this medium were used. Before protein extraction, cultures were microscopically observed to exclude the presence of microorganisms other than *Borrelia* and to appreciate bacterial growth at a concentration of 1×10^8^ bacteria/ml.

In order to verify the presence/absence of influence of bacterial growth phase on the identification based on protein profiles obtained by MALDI-TOF MS analysis, 4 cultures of *B*. *burgdorferi ss* B31 (ATCC 35210) were examined during early through late exponential phases of growth as well as in stationary phase. Each of the 4 cultures was harvested at different times (1, 2, 3, 4 and 7 days) of incubation at concentrations from 10^6^ to 10^8^ bacteria/ml. An aliquot (4 ml) of each of the harvested cultures at the different times of incubation was picked up and subjected to MALDI-TOF MS analysis after protein extraction (10 spots per concentration per culture) as described below.

### Sample preparation for MALDI-TOF MS

Preparation of bacterial strains for MALDI-TOF MS measurement was done as previously described [23] with some modifications. Briefly, for the protein extraction procedure a 3 ml aliquot of each *Borrelia* spp. culture was centrifuged at 13,000× g for 10 minutes and the supernatant was discarded. The pellet was re-suspended in 1 ml of sterile double-distilled water and re-centrifuged at 13,000× g for 10 minutes. The obtained pellet was suspended in 300 µl of sterile double-distilled water and then 900 µl of absolute ethanol were added. The suspension was centrifuged at 13,000× g for 10 minutes and the supernatant was discarded. After 40 minutes of air-drying to evaporate the ethanol residue, 30 µl of 70% formic acid and 30 µl of acetonitrile were added and the suspension was vortexed for 1 minute and centrifuged at 13,000× g for 2 minutes.

A polished steel MSP-96 target plate (Bruker Daltonics, Germany) was accurately cleaned with tap water in order to remove the residual matrix and then rinsed with sterile double-distilled water. Subsequently, the plate was deposited in 70% ethanol for 10 minutes, and finally dried with absorbent paper. After the formic acid/acetonitrile protein extraction, 1 µl of the supernatant was transferred on the plate in a unique deposition, and allowed to dry at room temperature for 10 minutes before being overlaid in a unique deposition with 1 µl of a satured α-cyano-4-hydroxy-cinnamic acid (HCCA) (2.5 mg) matrix solution (Bruker Daltonics, Germany) (previously resuspended in 250 µl of Organic Solvent, a solution of 50% acetonitrile and 2.5% tri-fluoro-acetic acid). After the matrix was air-dried for about 10 minutes at room temperature, the plate was immediately inserted into the instrument for analysis.

### MALDI-TOF MS analysis

The acquisition and the analysis of mass spectra were performed by a Microflex LT mass spectrometer (Bruker Daltonics, Germany) using the MALDI Biotyper Software package (version 3.0) with the reference database (version 3.1.2.0; 3,995 database entries, Bruker Daltonics, Germany) and default parameters setting (positive linear mode; laser frequency 60 Hz; ion source 1 voltage, 20 kV; ion source 2 voltage, 16.7 kV; lens voltage, 7.0 kV; mass range, 2,000 to 20,000 Da).

For each spectrum 240 laser shots in 40-shot steps from different positions of the sample spot were accumulated and analyzed (automatic mode, default setting).

In each experiment, the Bruker Bacterial Test standard (BTS) (Bruker Daltonics, Germany) for calibration was used according to the instructions of the manufacturer. Moreover, in each plate *Escherichia coli* (ATCC 8739) was used as a positive control and a non-inoculated-matrix solution as a negative control.

The Biotyper software compares each sample mass spectrum to the reference mass spectra present in the database using a pattern matching approach that is based on statistical multi-variant analysis and that takes into account peak positions and intensities. The software calculates an arbitrary unit score value comprised between 0 and 3.0 reflecting the similarity between sample and reference spectrum and finally displays the top ten matching database records. As specified by the manufacturer, identification scores ≥2.0 were accepted for a reliable identification at species level and score ≥1.7 and ≤2.0 for an identification at genus level. Scores < 1.7 were considered unreliable.

### MALDI-TOF MS database for *Borrelia* spp

For each of the 4 reference strains, 40 replicates were analyzed by MALDI-TOF MS. The respective obtained spectra were analyzed by the FlexAnalysis software (Version 3.0, Bruker Daltonics, Germany) to carry out “Smoothing” and “Baseline” and to select spectra with an intensity < 10^4^ arbitrary units and single spectra with peaks differing from the others. These selected spectra were removed and the remaining were used to calculate a reference Main Spectrum Profile (MSP) by the automated function of the Biotyper software (Biotyper MSP Creation Standard Method), as previously described [23]. MSPs were created by extracting information on peak position, intensity and frequency on the basis of an unbiased algorithm. The MSP spectra obtained were used for the MALDI Biotyper database supplementation.

### Statistical analysis

The same MALDI-TOF MS spectra previously selected for the database supplementation were imported into the ClinProTools software version 2.2 (Bruker Daltonics, Germany) to carry out statistical analysis. The software was used for visual comparison of the loaded spectra, as well as for identifying specific peaks discriminating among the analyzed strains.

The analysis was performed within the range 2,000 to 20,000 Da. First, the spectra for each of the investigated strains were loaded into the program and were automatically recalibrated. In order to compare individual strains, an equal number of spectra per strain was required: 25 spectra were selected and statistical testing of the datasets was performed on the basis of Principal Component Analysis (PCA) and the results were displayed in a three-dimensional score plot, automatically generated by the software.

The same spectra were then analyzed by 3 statistical algorithms which displayed a list of discriminating peaks for the analyzed spectra according to the selected algorithm. In particular, the algorithms were: Quick Classifier (QC) and Supervised Neural Network (SNN), that automatically give the highest value of “Recognition Capability (RC)” together with the highest value of “Cross Validation (CV)” with the highest number of peaks (from 1 to 25), and the Genetic Algorithm that gives a value of RC together with a value of CV based on the maximum number of peaks selected by the operator. The classifications obtained by the 3 models were compared on the basis of RC and CV parameters. The algorithm with the highest score of RC and the highest value of CV, taking into account also the number of the peaks utilized to obtain the model, was chosen to analyze the discriminating peaks that were displayed in a report. The presence/absence of each discriminating peak was evaluated by comparing the 4 average spectra automatically created from the 25 replicates of each reference strain analyzed.

### MALDI-TOF MS dendrogram

Cluster analysis (MSP dendrogram) was performed based on the comparison of the main spectra of the strains analyzed. In a first step, each main spectrum of the dataset was compared with each of the other main spectra resulting in a matrix of cross-wise identification scores. This matrix was used to calculate the distance values for each pair of main spectra. Based on these values a dendrogram was generated using the according function of the statistical toolbox of Matlab 7.1 (The MathWorks Inc.) integrated in the MALDI Biotyper software. The parameter settings were: “Distance Measure Euclidian” and “Linkage complete”. The linkage function is normalized according to the distance between 0 (perfect match) and 1,000 (no match).

## Results

**Figure 1:**
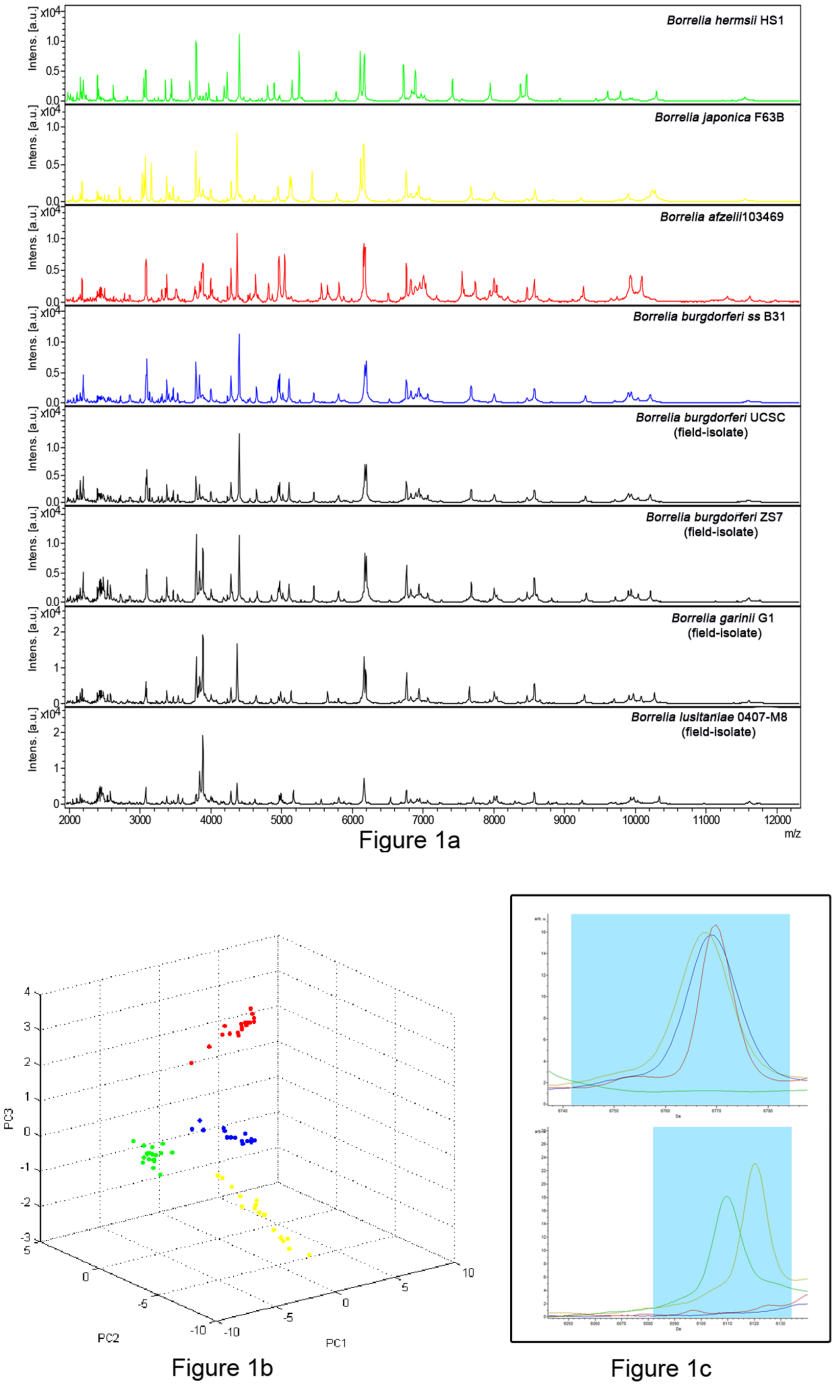
MALDI-TOF MS analysis of the reference strains of four *Borrelia* spp. and field isolates of both human and tick origin. (a): spectra obtained by MALDI-TOF MS analysis of the reference strains of four *Borrelia* spp. (*B*. *hermsii* HS1 in green, *B*. *japonica* F63B in yellow, *B*. *afzelii* 103469 in red, *B*. *burgdorferi ss* B31 in blue) and field isolates of both human (*B*. *burgdorferi ss* UCSC and *B*. *garinii* G1) and tick origin (*B. burgdorferi* ZS7 and *B. lusitaniae* MT 0407-M8), all in black. (b): three-dimensional plot of the spectra of the reference strains obtained by Principal Component Analysis (PCA) (dots of the same colour represent replicates of the same strain: *B*. *hermsii* HS1 in green, *B*. *japonica* F63B in yellow, *B*. *afzelii* 103469 in red, *B*. *burgdorferi ss* B31 in blue). (c): m/z window bins with the details of 2 among the 10 discriminating peaks within the 4 analyzed *Borrelia* reference strains (*B*. *hermsii* HS1 in green, *B*. *japonica* F63B in yellow, *B*. *afzelii* 103469 in red, *B*. *burgdorferi ss* B31 in blue).

Each of the 4 *Borrelia* spp. reference strains used in this study yielded a protein profile including unique peaks (Figure 1a), so that each *Borrelia* species tested gave a unique, species-specific MALDI-TOF MS profile. When compared with the available Bruker Daltonics database, *B*. *afzelii*, *B*. *hermsii*, and *B*. *japonica* protein profiles were found to be original, matching none of the existing profiles in the database (score value <1.3). In contrast, *B*. *burgdorferi ss* B31 (ATCC 35210) protein profile matched with the *B*. *burgdorferi ss* OE TWF profile (score value >2.3 for each of the 40 replicates) already present in the database, which included also *B*. *garinii* and *B*. *spielmanii*. No misidentification was found when all the *Borrelia* strains analyzed in this study were matched with the profiles obtained from spirochetes other than *Borrelia* (*Brachyspira* spp. and *Leptospira interrogans* [23]).

After selection by using the FlexAnalysis software of the 40 spectra obtained from each reference strain by MALDI-TOF MS, a minimum of 25 spectra was used to calculate a reference MSP spectrum through the automated function of the Biotyper software. The obtained 4 MSP reference spectra were deposited in the database for further blind identification of additional *Borrelia* strains.

In order to visualize and identify discriminatory peaks among the 4 different *Borrelia* species analyzed in this study, the spectra previously selected for the supplementation of the MALDI-Biotyper database were imported into the ClinProTools software. The statistical tool Principal Component Analysis (PCA) was applied to the analyzed datasets to visualize the homogeneity and heterogeneity of the protein spectra: PCA reduces the variables of a complex dataset generating a set of new variables called Principal Components (PC). In figure 1b a plot of the spectra in a three-dimensional space is reported showing the replicates of the spectra of the 4 different *Borrelia* spp. analyzed as 4 separate clusters.

The whole of the spectra within the same species were analyzed applying different algorithms available in the software. The QC model, chosen on the basis of an RC value of 100% and a CV value of 99.8%, displayed 10 discriminating peaks that are reported in Table 1. The combinations of the presence/absence of these peaks discriminated among the species analyzed in this study. As an example, m/z window bins of 2 among the 10 discriminating peaks within the *Borrelia* spp. analyzed are showed in Figure 1c.

**Table 1:** Patterns of 10 differentiating peaks based on the statistical analysis by the ClinProTools software of the 4 *Borrelia* species analyzed.

Peak mass (m/z) representing the protein size in Dalton				*Borrelia* spp.			
N.	Mass m/z [Da]	Start Mass m/z [Da]	End Mass m/z [Da]	*B. afzelii* 103469	*B. burgdorferi* B31	*B. hermsii* HS1	*B. japonica* F63B
1	3890,53	3879,53	3905,92	+	-	-	-
2	4005,69	3998,70	4014,17	+	+	-	+
3	4291,15	4278,05	4305,77	+	+	-	+
4	4376,47	4366,44	4385,10	+	-	-	+
5	4953,63	4931,65	4962,08	-	+	-	+
6	4974,68	4962,08	5005,23	+	+	-	-
7	6119,28	6081,80	6134,01	-	-	+	+
8	6181,13	6176,82	6193,10	+	+	-	-
9	6768,97	6741,62	6784,18	+	+	-	+
10	6944,05	6922,86	6955,01	-	+	-	+

The human isolate *B*. *burgdorferi ss* UCSC and other 4 field isolates (3 of human origin belonging to the species *B*. *burgdorferi*, *B*. *garinii* and *B*. *afzelii*, and 1 of tick origin belonging to the species *B*. *burgdorferi*) were identified by MALDI-TOF MS at species level with a score value >2.0. *B*. *lusitaniae* MT 0407-M8 (tick origin) was identified at genus level (score value 1.7–1.8 for all the replicates).

As an example, the spectra obtained for *B*. *burgdorferi ss* UCSC, *B*. *burgdorferi* ZS7, *B*. *garinii* G1, and *B*. *lusitaniae* MT 0407-M8 are shown in Figure 1a.

The spectra obtained by using 2 different lots of growth medium presented no differences between them for each analyzed strain.

The identification obtained by testing *B*. *burgdorferi ss* B31 (ATCC 35210) at different concentrations corresponding to different growth phases from early exponential phase to stationary phase was the same in all the conditions tested with a score >2.1 for all the replicates tested.

Moreover, each session was validated by external negative and positive controls: in all the experiments the negative control spots yielded no peaks or faint profiles which were not identifiable by the system, and the positive control spots yielded the expected *E*. *coli* identification with an identification score of 2.0–2.5.

**Figure 2:**
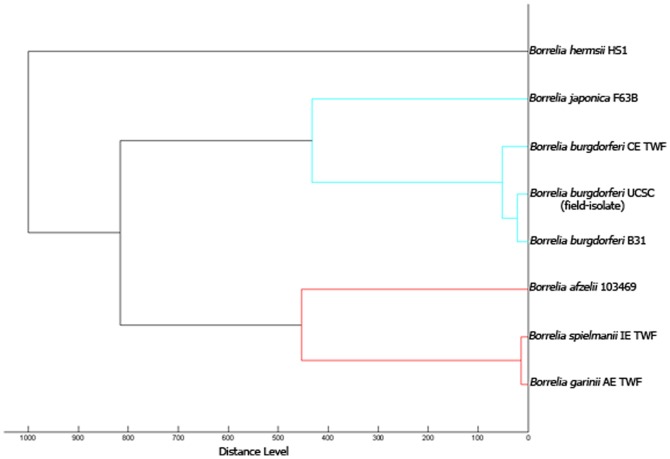
“Main Spectra Profiles”- based dendrogram of the *Borrelia* spp. used in this study to supplement the database.

The MSP spectra of the 4 *Borrelia* spp. reference strains and that of *B*. *burgdorferi ss* UCSC together with the MSP spectra of the 3 *Borrelia* species already present in the database (*B*. *burgdorferi ss* OE TWF, *B*. *garinii* and *B*. *spielmanii*) were used for the construction of a MSP spectra-based dendrogram (Figure 2).

## Discussion

This is the first study conducted about the application of MALDI-TOF MS to the identification of *Borrelia* species.

Despite improvements in prevention, diagnosis and treatment, LB is still the most common arthropod-borne bacterial disease in temperate regions of the northern hemisphere, with risk of infection associated with occupation (e.g. forestry work) and certain outdoor recreational activities (e.g. mushroom collecting) [3]. In our area a surveillance epidemiological study showed a sero-prevalence of LB of 0.55–2.27%, compatible with that of a non-endemic area [1]. In Europe, the annual number of LB cases is increasing in some areas, and tick vectors are expanding their range to higher altitudes and latitudes, suggesting that LB will remain an important health concern in the coming decades, especially in the light of economic, land-use and climate change predictions [3].

In a recent study [9] it was reported that among the *Borrelia* genospecies pathogenic for humans, *B*. *afzelii* and *B*. *garinii* are the predominant pathogens of LB in Europe followed by *B*. *burgdorferi ss*. For this reason, in our study we supplemented the Bruker Daltonics MALDI-TOF mass spectrometer database, originally including only the species *B*. *burgdorferi ss*, *B*. *spielmanii* and *B*. *garinii*, with the species *B*. *afzelii*. Moreover, it is well known that the quality and the reliability of the identification by MALDI-TOF MS depend on the quality and amount of reference spectra present in the database. For these reasons, we supplemented the commercially available database also with another reference strain of the agent of LB *B*. *burgdorferi ss* (B31 strain), with *B*. *hermsii*, that is the primary cause of tick-borne relapsing fever in western North America, and with *B*. *japonica*, a non-pathogenic species circulating in Asia, all available in the *Borrelia* strains collection of our laboratory.

In this study we demonstrated the ability of MALDI-TOF MS to correctly identify at species level field isolates of human and tick origin belonging to the species *B*. *burgdorferi ss*, *B*. *garinii*, and *B*. *afzelii* cultivated in BSK-II medium. As expected, the strain belonging to the species *B*. *lusitaniae* was identified only at genus level because this species was not included in the database. This result and the specific MALDI-TOF MS profile obtained for each *Borrelia* spp. reference strain suggest the possible application of this method to identify and discriminate among field isolates belonging to different species of the *B*. *burgdorferi sl complex*.

As compared to the molecular methods used for species delineation (such as DNA-DNA hybridization, Restriction Fragment Length Polymorphism and Multilocus Sequence Analysis) [10], MALDI-TOF MS is more rapid, less cumbersome and cheaper, in our own experience.

In this study the constructed dendrogram demonstrated the ability of MALDI-TOF MS to differentiate among *Borrelia* species and allowed to visualize intra-species similarities or variations (in the case of *B*. *burgdorferi ss*, for which 3 MSP spectra were available). The dendrogram reflects *Borrelia* taxonomy, showing that all species included in the *B. burgdorferi sl complex* cluster in a unique branch, while *B*. *hermsii* clusters separately.

The spectra obtained testing *B*. *burgdorferi ss* B31 (ATCC 35210) at different growth phases from early through late exponential phase until stationary phase showed that the identification by MALDI-TOF MS was not affected by *Borrelia* growth phase.

Recent surveys show that LB is still the most common arthropod-borne disease in temperate regions of the northern hemisphere and that the overall prevalence of LB may be stabilising, but its geographical distribution is expanding. Moreover, the increasing relevant health risk connected to LB has stimulated the research effort on all aspects of disease ecology and epidemiology [3]. It also emerges that standardized diagnosis is crucial to treating and combating LB in Europe; many authors agree that a concerted effort to improve surveillance is essential for monitoring this disease [2], [3], [32], [33].

In addition, much remains to be discovered about the factors affecting genospecific prevalence, transmission and virulence, although avoidance of tick bite still appears to be the most efficient preventive measure. Uniform, European-wide surveillance programmes (particularly on a social scale) and standardization of diagnostic tests and treatments are still urgently needed, especially in the light of climate change scenarios and land-use and socio-economic changes. Improved epidemiological knowledge will also aid the development of more accurate risk prediction models for LB [3].

In conclusion, in this study MALDI-TOF MS proved a useful tool that could be suitable for the application in the identification of *Borrelia* spp. both for diagnostic purposes and epidemiological surveillance. Moreover, our study suggests that in the future MALDI-TOF MS could prove useful to support the existence of different biotypes showing different protein profiles, possibly related to different pathogenic and virulence factors, to be further investigated by more sophisticated approaches in order to gain an insight into the biology of *Borrelia* spp.

## Supporting Information

Appendix S1 Peak lists of the 70 peaks included in the Main Spectrum Profiles (MSP) of each of the four reference strains of *Borrelia* spp., namely *B. afzelii* 103469, *B*. *burgdorferi ss* B31, *B*. *hermsii* HS1, *B*. *japonica* F63B analyzed in this study. The ratio m/z (Da), the intensity (%), and the frequency (%) with which each peak was present in the different replicates composing the MSP are indicated. The weight (%) with which each peak was considered for subsequent identification based on the newly created MALDI-TOF database.
(PDF)
