# Supplementary material for: Identification of Borrelia Species after Creation of an In-House MALDI-TOF MS Database
Source: PLoS One. 2014 Feb 12;9(2):e88895. doi: 10.1371/journal.pone.0088895 (PMC3923052; doi:10.1371/journal.pone.0088895)
Supplement: Appendix S1 — Peak lists of the 70 peaks included in the Main Spectrum Profiles (MSP) of each of the four reference strains of Borrelia spp., namely B. afzelii 103469, B. burgdorferi ss B31, B. hermsii HS1, B. japonica F63B analyzed in this study. The ratio m/z (Da), the intensity (%), and the frequency (%) with which each peak was present in the different replicates composing the MSP are indicated. The weight (%) with which each peak was considered for subsequent identification based on the newly created MALDI-TOF database. (PDF) [file pone.0088895.s001.pdf]

MALDI Biotyper MSP Peak List Editor. Version: 3.1.66.0<br><br>*Borrelia afzelii strain* 103469			
**m/z [Da]**	**Intensity [%]**	**Weight [%]**	**Frequency [%]**
3087,73	65,64	100,00	100,0
3165,25	7,06	100,00	83,3
3313,86	18,48	100,00	100,0
3365,63	14,28	100,00	100,0
3384,47	28,23	100,00	100,0
3511,26	14,90	100,00	100,0
3544,26	8,00	100,00	76,7
3602,25	5,58	100,00	67,7
3795,90	49,80	100,00	96,7
3848,27	25,84	100,00	100,0
3875,44	40,24	100,00	86,7
3888,77	66,91	100,00	100,0
4005,72	24,66	100,00	100,0
4022,37	14,08	100,00	93,3
4057,09	5,03	100,00	66,7
4239,37	16,36	100,00	100,0
4290,67	40,17	100,00	100,0
4308,17	20,18	100,00	63,3
4376,72	89,65	100,00	100,0
4397,78	10,25	100,00	93,3
4530,94	9,97	100,00	66,6
4555,47	11,08	100,00	100,0
4615,63	15,59	100,00	80,0
4636,91	37,61	100,00	100,0
4797,79	5,57	100,00	63,3
4818,86	26,19	100,00	100,0
4875,99	13,49	100,00	96,7
4971,88	78,02	100,00	100,0
5048,20	67,35	100,00	100,0
4136,26	6,31	100,00	63,3
5569,12	23,89	100,00	76,7
5813,87	23,95	100,00	100,0
5884,36	7,12	100,00	70,0
6100,54	4,25	100,00	66,7
6144,50	21,68	100,00	90,0
6174,11	97,66	100,00	100,0
6224,18	11,73	100,00	83,3
6512,99	9,24	100,00	66,7
6769,16	45,00	100,00	100,0
6833,52	24,10	100,00	100,0
6959,60	15,56	100,00	96,7
7014,09	21,52	100,00	100,0
7043,89	10,74	100,00	96,7
7196,29	8,63	100,00	70,0
7716,60	15,91	100,00	96,7
7740,36	30,01	100,00	100,0
7868,69	3,95	100,00	63,3
7946,05	10,06	100,00	96,7
8007,77	27,52	100,00	100,0
8043,71	16,50	100,00	100,0
8112,87	5,45	100,00	63,3

MALDI Biotyper MSP Peak List Editor. Version: 3.1.66.0<br><br>*Borrelia afzelii strain* **103469**			
**m/z [Da]**	**Intensity [%]**	**Weight [%]**	**Frequency [%]**
8203,69	6,29	100,00	76,7
8475,52	20,53	100,00	100,0
8548,03	9,96	100,00	93,3
8576,97	30,85	100,00	100,0
8610,81	10,22	100,00	100,0
8788,40	8,55	100,00	70,0
9239,96	12,51	100,00	90,0
9267,89	22,85	100,00	100,0
9662,25	4,96	100,00	66,7
9746,20	10,67	100,00	93,3
9902,94	16,29	100,00	70,0
9937,87	44,69	100,00	100,0
10012,75	10,74	100,00	97,7
10092,79	40,89	100,00	100,0
10162,59	5,80	100,00	73,3
11620,88	9,78	100,00	73,3
13623,94	7,90	100,00	73,3
13661,41	13,54	100,00	96,7
13910,58	4,94	100,00	63,3

MALDI Biotyper MSP Peak List Editor. Version: 3.1.66.0<br><br>*Borrelia burgdorferi strain* B31			
**m/z [Da]**	**Intensity [%]**	**Weight [%]**	**Frequency [%]**
3097,87	49,37	100,00	100,0
3136,09	12,06	100,00	100,0
3172,29	7,80	100,00	100,0
3263,67	4,02	100,00	91,7
3316,51	9,66	100,00	100,0
3384,98	22,30	100,00	100,0
3408,27	6,35	100,00	100,0
3450,47	4,87	100,00	91,7
3472,60	16,60	100,00	100,0
3537,50	12,95	100,00	100,0
3628,96	2,14	100,00	83,3
3796,04	45,63	100,00	95,8
3844,04	26,84	100,00	100,0
3889,79	8,86	100,00	100,0
4007,15	17,76	100,00	100,0
4084,60	2,77	100,00	91,7
4239,92	7,59	100,00	100,0
4291,15	35,61	100,00	100,0
4309,57	7,50	100,00	79,2
4366,78	4,77	100,00	95,8
4404,73	100,00	100,00	100,0
4427,89	6,27	100,00	87,5
4556,13	6,43	100,00	100,0
4629,03	5,27	100,00	91,7
4653,01	20,31	100,00	100,0
4863,29	6,91	100,00	95,8
4955,79	30,13	100,00	95,8
4975,48	40,53	100,00	100,0
5024,46	11,17	100,00	100,0
5110,45	39,97	100,00	100,0
5136,15	6,76	100,00	83,3
5187,28	2,08	100,00	87,5
5264,07	4,95	100,00	95,8
5460,18	15,77	100,00	100,0
5726,30	1,81	100,00	83,3
5806,99	17,10	100,00	100,0
5885,89	7,70	100,00	100,0
6201,98	65,15	100,00	100,0
6529,70	9,39	100,00	100,0
6738,82	3,10	100,00	87,5
6769,42	36,13	100,00	100,0
6833,10	15,06	100,00	100,0
6902,96	11,06	100,00	100,0
6944,46	22,07	100,00	100,0
6976,28	10,10	100,00	100,0
7070,61	11,37	100,00	100,0
7253,14	5,44	100,00	83,3
7652,64	7,68	100,00	100,0
7685,00	30,89	100,00	100,0
7792,68	3,18	100,00	100,0
8010,31	20,23	100,00	100,0

MALDI Biotyper MSP Peak List Editor. Version: 3.1.66.0<br><br>*Borrelia burgdorferi strain* B31			
**m/z [Da]**	**Intensity [%]**	**Weight [%]**	**Frequency [%]**
8063,67	2,87	100,00	95,8
8347,40	2,33	100,00	95,8
8475,11	9,07	100,00	100,0
8542,69	6,64	100,00	100,0
8578,51	30,32	100,00	100,0
8610,04	7,06	100,00	87,5
9255,77	5,61	100,00	79,2
9298,99	13,26	100,00	100,0
9720,00	4,63	100,00	95,8
9906,39	20,45	100,00	100,0
9945,51	20,30	100,00	100,0
10042,62	7,19	100,00	100,0
10215,98	20,52	100,00	100,0
10272,91	3,82	100,00	83,3
11606,18	6,79	100,00	100,0
11763,02	3,66	100,00	91,7
13051,40	4,19	100,00	95,8
13657,73	3,90	100,00	100,0
13943,70	2,98	100,00	100,0

MALDI Biotyper MSP Peak List Editor. Version: 3.1.66.0<br><br>*Borrelia hermsii strain* HS1			
**m/z [Da]**	**Intensity [%]**	**Weight [%]**	**Frequency [%]**
3055,58	28,86	100,00	77,1
3085,37	37,98	100,00	94,3
3264,59	6,69	100,00	77,1
3363,37	27,26	100,00	100,0
3444,53	33,78	100,00	100,0
3487,85	8,61	100,00	97,1
3709,46	25,52	100,00	97,1
3796,79	46,96	100,00	88,6
3851,06	13,23	100,00	100,0
3891,89	14,13	100,00	97,1
3936,72	10,37	100,00	85,7
3972,65	32,19	100,00	100,0
4084,66	7,23	100,00	65,7
4192,88	19,64	100,00	100,0
4233,91	45,27	100,00	100,0
4406,58	94,91	100,00	100,0
4469,59	26,35	100,00	100,0
4561,83	7,72	100,00	85,7
4649,75	10,21	100,00	48,6
4680,42	4,36	100,00	60,0
4725,54	12,53	100,00	100,0
4807,59	41,71	100,00	100,0
4897,27	37,68	100,00	100,0
4977,22	17,17	100,00	100,0
5027,49	3,48	100,00	60,0
5123,44	13,24	100,00	68,6
5151,62	37,39	100,00	100,0
5252,47	51,99	100,00	100,0
5288,67	5,78	100,00	62,9
5621,15	6,67	100,00	65,7
5691,27	4,31	100,00	62,9
5776,85	33,32	100,00	100,0
5895,87	15,96	100,00	77,1
5918,95	12,01	100,00	42,9
6111,68	80,29	100,00	100,0
6170,93	74,00	100,00	100,0
6697,46	7,79	100,00	60,0
6725,57	54,47	100,00	94,3
6847,41	27,01	100,00	100,0
6890,93	44,19	100,00	100,0
6973,59	40,28	100,00	100,0
7017,97	16,62	100,00	100,0
7195,96	11,41	100,00	77,1
7386,35	8,79	100,00	80,0
7417,70	37,73	100,00	100,0
7595,48	12,38	100,00	42,9
7772,65	13,33	100,00	51,4
7949,84	44,99	100,00	100,0
8379,85	26,30	100,00	100,0
8433,23	12,67	100,00	60,0
8463,30	51,52	100,00	100,0

MALDI Biotyper MSP Peak List Editor. Version: 3.1.66.0<br><br>*Borrelia hermsii strain* HS1			
**m/z [Da]**	**Intensity [%]**	**Weight [%]**	**Frequency [%]**
8613,50	5,96	100,00	65,7
8820,85	4,59	100,00	62,9
8933,87	32,43	100,00	100,0
9446,54	12,74	100,00	100,0
9610,70	28,34	100,00	100,0
9672,50	3,00	100,00	45,7
9789,32	24,07	100,00	100,0
9924,41	6,99	100,00	82,9
9953,40	10,06	100,00	97,1
10097,38	4,25	100,00	48,6
10232,93	8,89	100,00	62,9
10298,89	23,95	100,00	100,0
11234,80	6,87	100,00	45,7
11376,09	2,76	100,00	51,4
11548,34	18,58	100,00	100,0
11781,94	10,71	100,00	57,1
13676,86	8,78	100,00	100,0
13939,00	16,08	100,00	94,3
14027,64	8,21	100,00	88,6

MALDI Biotyper MSP Peak List Editor. Version: 3.1.66.0<br>*Borrelia japonica strain* F63B			
**m/z [Da]**	**Intensity [%]**	**Weight [%]**	**Frequency [%]**
3056,68	35,16	100,00	100,0
3083,28	58,42	100,00	100,0
3162,63	49,33	100,00	100,0
3261,44	3,44	100,00	100,0
3300,63	9,66	100,00	100,0
3384,62	37,67	100,00	100,0
3419,64	14,24	100,00	94,1
3472,82	25,75	100,00	100,0
3547,18	11,75	100,00	100,0
3795,45	52,01	100,00	100,0
3843,57	35,25	100,00	100,0
3890,84	18,81	100,00	100,0
3911,17	7,28	100,00	82,4
3936,62	3,78	100,00	100,0
3969,03	4,19	100,00	94,1
4006,38	21,81	100,00	100,0
4172,64	3,59	100,00	100,0
4238,49	8,49	100,00	100,0
4296,49	39,39	100,00	100,0
4376,12	95,41	100,00	100,0
4439,38	9,12	100,00	100,0
4555,24	9,11	100,00	100,0
4622,39	26,45	100,00	100,0
4653,86	3,43	100,00	94,1
4724,08	3,69	100,00	88,2
4893,00	12,71	100,00	100,0
4952,19	40,86	100,00	100,0
5083,59	8,51	100,00	100,0
5124,42	44,10	100,00	88,2
5434,95	47,38	100,00	100,0
5459,43	5,16	100,00	82,4
5499,37	2,31	100,00	88,2
5602,14	3,10	100,00	82,4
5716,62	3,38	100,00	100,0
5785,46	22,31	100,00	100,0
5840,30	4,12	100,00	100,0
6120,79	70,72	100,00	100,0
6166,40	96,79	100,00	100,0
6519,82	24,59	100,00	100,0
6736,51	5,17	100,00	100,0
6767,59	51,54	100,00	100,0
6830,53	16,91	100,00	100,0
6909,89	16,23	100,00	100,0
6943,03	27,46	100,00	100,0
6986,12	3,33	100,00	94,1
7089,39	5,76	100,00	100,0
7651,88	7,60	100,00	94,1
7682,31	30,04	100,00	100,0
7797,28	4,69	100,00	100,0
7876,36	2,68	100,00	100,0
7951,81	3,85	100,00	100,0

MALDI Biotyper MSP Peak List Editor. Version: 3.1.66.0<br><br>*Borrelia japonica strain* **F63B**			
**m/z [Da]**	**Intensity [%]**	**Weight [%]**	**Frequency [%]**
8008,77	24,08	100,00	100,0
8044,88	7,42	100,00	100,0
8341,51	3,58	100,00	100,0
8471,92	7,86	100,00	100,0
8552,86	6,11	100,00	100,0
8588,41	29,46	100,00	100,0
8773,61	3,49	100,00	88,2
8839,89	7,57	100,00	100,0
9239,82	18,11	100,00	100,0
9777,92	4,72	100,00	100,0
9899,71	22,46	100,00	100,0
9963,19	3,26	100,00	100,0
10163,15	5,70	100,00	100,0
10244,43	23,23	100,00	100,0
10284,23	20,54	100,00	100,0
11564,11	8,28	100,00	100,0
13030,39	9,27	100,00	100,0
13653,29	4,44	100,00	100,0
13810,75	2,82	100,00	100,0
